# Deletion of the CD2 Gene in the Virulent ASFV Congo Strain Affects Viremia in Domestic Swine, but Not the Virulence

**DOI:** 10.3390/ani13122002

**Published:** 2023-06-15

**Authors:** Andrey Koltsov, Sergey Krutko, Natalia Kholod, Mikhail Sukher, Sergey Belov, Alexey Korotin, Galina Koltsova

**Affiliations:** Federal Research Centre for Virology and Microbiology, Academician Bakoulov Street 1, 601125 Volginsky, Russia; kolcov.andrew@gmail.com (A.K.); natkholod@yandex.ru (N.K.);

**Keywords:** ASF, ASFV, recombinant virus, CD2v, EP402R

## Abstract

**Simple Summary:**

African swine fever (ASF) causes serious economic losses to the global pig industry. Unfortunately, there is currently no effective and safe vaccine, although its development is actively continuing. To date, experimental vaccines based on the use of live attenuated strains of the ASF virus (ASFV) obtained by the deletion of viral genes responsible for virulence are the most effective. However, deletion of the same gene in different ASFV isolates can lead to different results. In our work, we have shown that, unlike some other isolates, the deletion of the CD2 gene from the genome of the virulent Congo strain does not lead to its weakening, although it affects the onset of ASF infection in domestic pigs.

**Abstract:**

African swine fever (ASF) is an infectious disease that causes the most significant losses to the pig industry. One of the effective methods for combating this disease could be the development of vaccines. To date, experimental vaccines based on the use of live attenuated strains of the ASF virus (ASFV) obtained by the deletion of viral genes responsible for virulence are the most effective. Deletion of the EP402R gene encoding a CD2-like protein led to the attenuation of various strains of the ASFV, although the degree of attenuation varies among different isolates. Here we have shown that the deletion of the EP402R gene from the genome of a high-virulent Congo isolate did not change either the virulence of the virus or its ability to replicate in the swine macrophage cell cultures in vitro. However, in vivo, animals infected with ΔCongo-v_CD2v had a delay in the onset of the disease and viremia compared to animals infected with the parental strain. Thus, deletion of the CD2 gene in different isolates of the ASFV has a different effect on the virulence of the virus, depending on its genetic background.

## 1. Introduction

African swine fever (ASF) is a viral hemorrhagic disease of domestic pigs, of which the mortality rate reaches 100% [1,2,3]. After the ASF genotype II virus was imported from East Africa to Georgia [4], it began to circulate in Eastern Europe (since 2007), in the European Union (since 2014), in Asia (since 2018), in America (since 2021) and Oceania (since 2020) (OIE).

ASFV is a large DNA-containing cytoplasmic virus that belongs to the *Asfarviridae* family [5]. Genotyping based on the B646L gene (encoding the p72 protein) made it possible to identify 24 genotypes of the ASF virus [6,7]. ASFV encodes more than 150 ORFs, the function of most of which is still unknown [5]. Understanding the functions of viral proteins is crucial for the rational development of vaccines and antiviral drugs. Gene deletions can be used both to study gene functions and to obtain a live-attenuated ASF vaccine using genetic engineering methods. One of the genes whose deletion can lead to a reduction in the virulence of ASFV is CD2v [8].

The CD2v protein of the ASFV (EP402R, or 8-DR) is a hemagglutinin, which is responsible for the characteristic formation of erythrocyte rosettes around ASFV-infected cells; it also contributes to the development of erythrocyte-associated viremia associated with the formation of protective immunity [9,10,11,12]. It has been demonstrated that the CD2v protein may play a role in the pathogenesis of ASF, in enfeebling the immune response and enhancing the replication of the virus in animals [13,14,15,16,17].

We have recently shown that CD2v proteins (encoded by the EP402R gene) and/or C-type lectin (encoded by the EP153R gene) of the ASF virus are necessary to determine serological specificity in the hemadsorption inhibition reaction (HAI), and may be important for protecting pigs from infection with homologous ASFV [18,19,20]. Previously, several strains of ASF virus with deletion of the gene encoding the CD2v protein were obtained (BA71, Malawi, Georgia, Congo-a (Congo attenuated) and ASFV-Kenya-IX-1033); however, published data on the role of this protein in the virulence of the virus and in protection against ASF are contradictory [8,16,20,21,22,23]. This contradiction may be due to the fact that the ASF virus exhibits a high degree of genetic variability, so studying CD2v in strains of different genotypes and/or serotypes could provide more information to understand its function.

A pair of virulent Congo-v strain (K49) and attenuated Congo-a strain (KK262) is used by us as a well-characterized model for studying both the functions of ASFV genes and the protective immune response against ASF [20,24]. Both strains belong to genotype I and serogroup 2 based on p72 and CD2v sequences, respectively [18,25]. In addition, these strains were assigned to seroimmunotype 2 in accordance with the results of animal vaccination/challenge experiments and serological tests (HAI) [26,27].

Previously, we demonstrated that the deletion of CD2v in the attenuated Congo-a strain did not affect the ability of this mutant to replicate in cell cultures; however, immunization of animals with this virus did not lead to the formation of protective immunity in response to infection with the homologous virulent ASFV Congo-v strain [20]. In this study, a deletion of the EP402R gene was introduced into the highly virulent ASFV Congo-v strain to find out whether this deletion leads to a decrease in virulence and replication in vivo in domestic pigs.

## 2. Materials and Methods

### 2.1. Cell Cultures and Viruses

A4C2/9 k cells (*Sus scrofa domesticus)* [28] and ASF Congo-v virulent virus (strain K49, genotype I, serogroup 2) were obtained from the collection of the FRC of Virology and Microbiology (Volginsky, Russia). The K49 strain was originally isolated from a domestic pig in the Democratic Republic of the Congo [29]. A4C2/9k was obtained at the Federal Research Centre for Virology and Microbiology from a transplanted hybrid cell line of SPEV TK with porcine splenocytes (A4C2) [30]. Primary cultures of porcine macrophages were prepared from blood using a medium for separating lymphocytes. The obtained cells were cultured in 96-well (1–3 × 10^6^ cells/well) or 48-well plates (4 × 10^6^ cells/well) (Corning, New York, NY, USA) in media containing RPMI 1640 with the addition of 10% (*v*/*v*) fetal bovine serum (Gibco), 30% (*v*/*v*) plasma, and antibiotic (Gibco) in a CO_2_ incubator (temperature 37 °C, 5% CO_2_) for 24 h. The adherent cells were washed with the same medium and used in experiments after 36–48 h.

Viruses were titrated in 96-well plates based on the presence of cytopathic effect (CPE) in primary cultures of porcine macrophages. Titers were expressed as the average infectious dose of tissue culture (TCID50) using the Reed–Munch method [31].

The growth curves of the parental Congo-v virus and its mutant ΔCongo-v_CD2v were measured after the infection of primary cultures of porcine macrophages in 48-well plates. Cultures were infected with viruses at MOI = 0.1 or 1; then, after 1 h of adsorption, the virus was removed; the cells were washed with PBS and incubated in a macrophage medium (37 °C, 5% CO_2_). The cell suspension was collected at 0, 24, 48, 72 and 96 h after infection (hpi) and stored at a temperature of −50 °C. These viral preparations were then used to determine TCID50/mL titers in primary cultures of porcine macrophage cells.

### 2.2. Construction of ΔCongo-v_CD2v Recombinant Virus

ΔCongo-v_CD2v was obtained from the virulent ASF Congo-v virus (K49, SG2), using a recombination cassette consisting of the reporter gene GFP (under the control of the viral promoter p72), flanked by two recombination arms, as described earlier [18,20]. A4C2/9k cells were transfected with recombination vectors and infected with the parent virus (at MOI = 5) in 6-well plates.

The recombinant virus was selected by the method of limited dilutions in primary cultures of porcine macrophages using fluorescence microscopy. The purity of the selected virus was confirmed using real-time PCR with primers specific to the EP402R gene or to the recombination site as described earlier [20]. The sequence accuracy of the recombinant virus was confirmed by sequencing a DNA fragment (position 72,193–74,538 in the genome of ASFV K49, GenBank MZ202520.1). Sequencing was done using the Sanger method on a 3130xl Genetic Analyzer (Thermo Fisher, Waltham, MA, USA).

The absence of CD2v gene expression in cells infected with ΔCongo-v_CD2v was confirmed in hemadsorption assay (HA) according to the standard protocol [19]. In short, primary macrophages of pigs were infected with the parental Congo-v virus or recombinant ΔCongo-v_CD2v virus (MOI = 0.1) in the presence of porcine erythrocytes. HA was evaluated using light microscopy 2–6 days after infection.

### 2.3. Animal Experiments

All animal procedures were conducted in accordance with Russian legislation on the protection of animals used for scientific purposes, and the experiments were approved by the Research Ethics Committee of the Federal Research Center of Virology and Microbiology, Russia (№ 06, 23 July 2021).

Piglets not vaccinated against any infections were received from a commercial pig farm (Russia). A total of 18 white pigs (males/females) aged 2–2.5 months (weighing 15–18 kg) were obtained. The piglets were randomly divided into three groups. Each group of animals was kept in isolated rooms throughout the experiment. After a 1-week acclimatization period, pigs from group 1 (n = 5) were infected intramuscularly with Congo-v virus. Pigs from group 2 (n = 8) were also infected intramuscularly with the ΔCongo-v_CD2v virus. Both viruses were injected at a dose of 10^3^ TCID50. Pigs from group 3 (n = 5) were left as controls and were inoculated with a PBS buffer.

The pigs’ rectal body temperature and clinical signs were measured daily throughout the experiment. Clinical evaluation of ASF was performed in 4 different categories (behavior, neurologic signs, defecation and body temperature). The clinical signs were assigned numerical values based on the severity and significance as described in [32]. The total score was recorded as a clinical score. Clinical scores were recorded daily for each pig.

Time to death and survival were recorded as described earlier [19]. The day-to-fever value corresponded to the first-day post-challenge when rectal body temperature was recorded to rise above 40 °C. To assess viremia, blood samples were taken from the jugular vein 0, 1, 3, 5 and 7 days post-inoculation (dpi). Five tissues (lung, liver, spleen, mesenteric and submandibular lymph nodes) were taken from animals during autopsy and frozen at −70 °C.

The viral load in the blood and organ samples of infected animals was assessed using titration in the primary porcine macrophage cell culture, as described above.

## 3. Results

### 3.1. Construction of ΔCongo-v_CD2v Recombinant Virus

**Figure 1:**
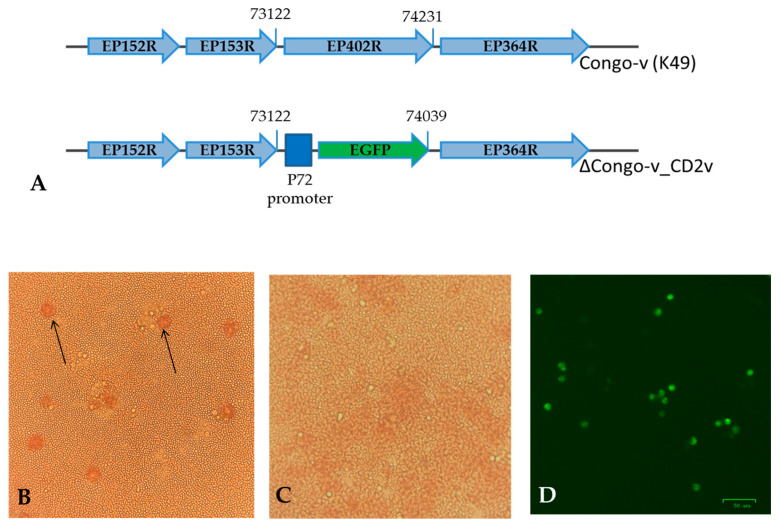
Construction of the ΔCongo-v_CD2v virus. A schematic diagram representing the target site for deletion of the EP402 gene in the Congo-v genome (**A**). Hemabsorption assays (HA) in primary porcine macrophages infected with parental Congo-v virus (**B**) or ΔCongo-v_CD2v (**C**) 2 days after inoculation. The arrows point to the erythrocyte rosettes around ASFV-infected cells (**B**). Fluorescent microscopy of primary swine macrophages infected with ASFV ΔCongo-v_CD2v (MOI = 0.1) 2 days after inoculation (**D**).

To study the function of CD2v in virulent variants of the ASF virus, a recombinant Congo virus (Congo-v) with deletion of the EP402R gene was constructed. This ΔCongo-v_CD2v virus was obtained by homologous recombination using a construct containing two “arms” of recombination and a GFP reporter gene under the ASFV promoter p72 (Figure 1A). The resulting recombinant virus ΔCongo-v_CD2v contained a deletion of the CD2 gene with a size of 1110 bp (the positions of nucleotides in the genome are 73,122–74,231) and an insertion of the GFP gene with a size of 917 bp compared to the original Congo-v strain (Figure 1A).

The recombinant virus was obtained as a result of selection during nine rounds of limiting dilutions in primary cultures of pig macrophages. The absence of a wild-type virus, as well as the presence of a recombination event in the final preparation of the recombinant virus, was confirmed using PCR. Real-time PCR with primers specific to the EP402R gene showed the absence of the target product. PCR analysis with primers flanking the recombination site revealed an expected fragment of 2.35 kb in size in the case of the parent Congo-v virus and a fragment of 2.15 kb in size in the case of the recombinant ΔCongo-v_CD2v virus. The replacement of the EP402R gene with the GFP gene and the absence of additional mutations in the fragment ΔCongo-v_CD2v genome were also confirmed by sequencing.

Hemadsorption in macrophage cell cultures infected with recombinant ΔCongo-v_CD2v was not observed, which also confirmed the absence of CD2 protein due to the deletion of the EP402R gene (Figure 1C). Moreover, cells infected with the ΔCongo-v_CD2v virus express the green fluorescent protein gene (Figure 1D).

### 3.2. In Vitro Growth Kinetics of ΔCongo-v_CD2v in Swine Macrophage Cell Cultures

**Figure 2:**
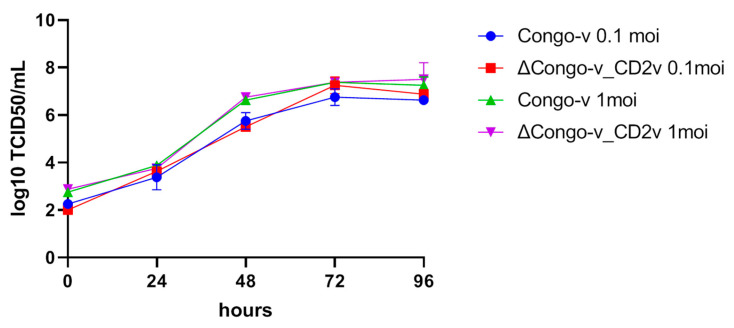
In vitro growth kinetics of Congo-v (K49) virus and recombinant ΔCongo-v_CD2v virus in primary swine macrophage cultures. Multistep growth curves are given as in log10 TCID50/mL. The analysis was carried out using Graphpad Prism software (version 8.0.1).

To study the role of the CD2v protein in the replication of ASFV, in vitro multistage growth curves of viruses in primary cultures of pig macrophages were constructed. The growth curves of the ASF virus ΔCongo-v_CD2v and the parental virulent ASF virus Congo-v were measured in cells infected with viruses with an MOI equal to 1 or 0.1. The multiplicity of infection equal to 0.1 was used to ensure multiple replication cycles during the experiment. Infected cells were used to determine TCID50/mL titers in primary cultures of swine macrophage cells. The analysis of the data obtained showed that no differences in the growth of viruses were detected until the end of the observation period (96 hpi) (Figure 2). In addition, we determined that the original strain K49 and the recombinant strain were effectively replicated in the primary culture of pig bone marrow, accumulating in the titer up to 7–8 Lg TCID50/mL. As expected, no hemadsorption was observed during the replication of the recombinant ΔCongo-v_CD2v virus in primary cultures of porcine macrophages (in the presence of porcine erythrocytes), unlike the replication of the Congo-v virus.

### 3.3. Replication and Virulence In Vivo in Susceptible Animals

**Figure 3:**
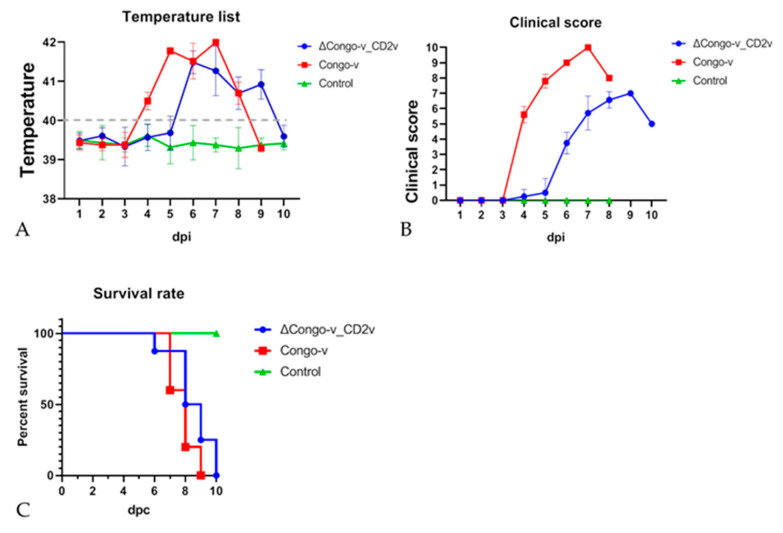
Body temperature (**A**), clinical signs (**B**) and lethality kinetics (**C**) in pigs inoculated with ASFV Congo-v strain (K49) and recombinant strain ΔCongo-v_CD2v. The dashed line represents the fever-cutoff (40 °C) (A). Pigs were inoculated with PBS (green line), Congo-v (strain K49) (red line) and recombinant strain ΔCongo-v_CD2v (blue line). Data on clinical assessment and body temperature are given in the form of average values (and SD) for each of the groups. The analysis was performed using Graphpad Prism software (version 8.0.1).

The viral pathogenesis and virulence of the recombinant strain ASFV ΔCongo-v_CD2v were studied in comparison with the parental strain Congo-v in an animal experiment. To do this, two groups of animals were inoculated intramuscularly with either a parent or recombinant virus at a dose of 10^3^ TCID50. Pigs from group 3 were inoculated with PBS and were used as a control in the experiment. Animal observations showed that all control pigs had no clinical signs associated with the disease (Figure 3A,B). After infection with ASFV Congo-v and ASFV ΔCongo-v_CD2v, all animals developed typical clinical symptoms of ASF, including hyperthermia, depression, anorexia and skin cyanosis (Figure 3A,B). There was no bleeding from the nose or rectum. We noted small changes at the onset of the disease; the increase in temperature and the appearance of clinical signs in animals inoculated with the recombinant strain began a day later than in animals inoculated with the parental strain (Figure 3A,B). Moreover, clinical signs in animals infected with the ΔCongo-v_CD2v strain were lower compared to animals infected with the Congo-v strain (Figure 3B).

All pigs infected with both recombinant and parental viruses died or were found in a dying state and euthanized within 6–10 days (Figure 3C).

**Figure 4:**
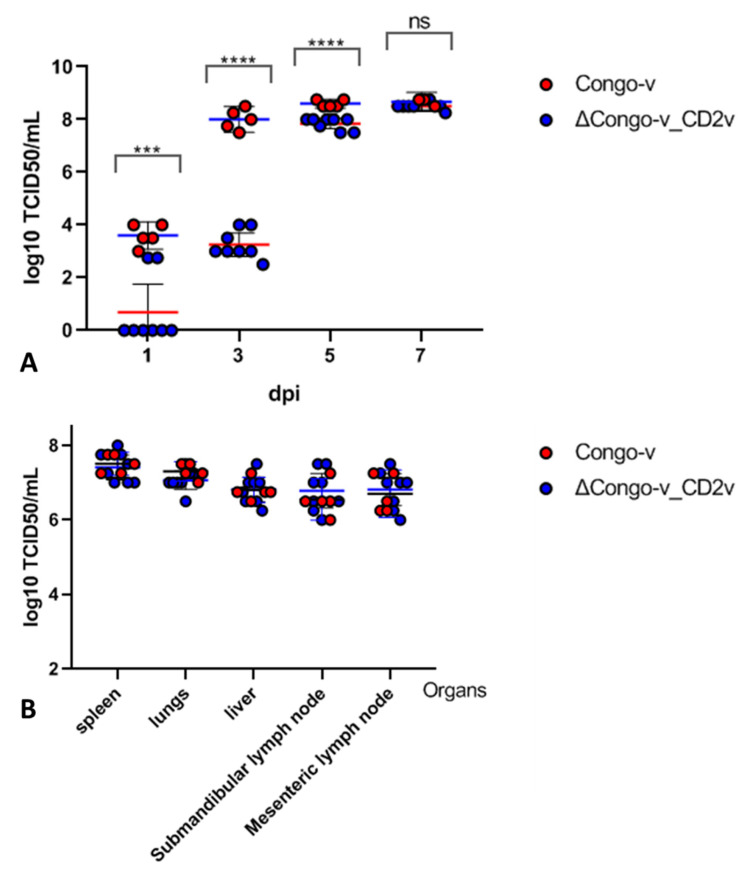
Daily viral loads in blood (**A**) and post-mortem viral loads in tissues (**B**) in pigs infected with ASFV Congo-v or ASFV ΔCongo-v_CD2v strains. The data on viral load are presented in the form of average values in log10 TCID50 as mean with 95% CI. The analysis was performed using Graphpad Prism software (version 8.0.1). *p*-values were determined using one-way ANOVA (ns, *p* > 0.05; *** *p* < 0.001; and **** *p* < 0.0001).

To understand the reasons for this delay at the onset of the disease, we investigated the viral load in the blood and organs of pigs (Figure 4). We found that in most animals infected with the recombinant strain ΔCongo-v_CD2v, the titers of the virus in the blood on the 1st day after infection were not detected; only two animals had 2.75 log10 TCID50 of the ASFV in their blood (Figure 4A). At the same time, animals infected with the parental Congo-v strain had virus titers of 3–4 log10 TCID50. On the third day after infection, the titer of the virus in animals infected with the parental strain reached 8 log10 TCID50, while the titer of the virus in pigs infected with the recombinant virus was only 2.5–4 log10 TCID50 (Figure 4A). However, there were no significant differences in viremia at later stages of infection (5–7 dpi) in animals of both groups.

Studies of the organs of pigs infected with recombinant or parental virus have not shown a significant difference in pathological changes. In addition, viral titers of the spleen, liver, lungs and lymph nodes (submandibular and mesenteric) were comparable in two groups (Figure 4B). Due to the fact that the organs were collected only after the death of the animals, we had no information about the spread of the virus in the organs of infected animals in the early stages of infection.

A summary of the animal experiment is given in Table 1. There were no significant differences in mortality, but a statistically significant difference in the time-to-fever was found for groups of animals infected with a parent or recombinant virus. In addition, there was a significant difference in viral load in blood at the early stage of infection, which then disappeared by the late stage of the disease.

## 4. Discussion

African swine fever causes huge economic losses to the pig industry all over the world. To date, no effective and safe vaccines have been developed, but the greatest success has been achieved with the use of live attenuated viruses. These non-pathogenic viruses can be identified in nature, as well as obtained in the laboratory by deletion of the genes responsible for virulence.

Along with the genes I177L, 9GL, UK, I226R, A137R and MGF360/MGF505 [33,34,35,36,37,38], the EP402R gene (encoding the CD-like protein) is one of the genes whose deletion can affect the virulence of the ASF virus [8]. Since its discovery, the CD2v protein of the ASFV has been at the center of research on its role in virus replication and pathogenesis. It has been shown that the CD2v protein is responsible for mediating hemadsorption [9,10], is important for virus replication in tick cells [14], and participates in the formation of a protective immune response [16,19]. The interaction of CD2v with the AP-1 host protein has been described, although the importance of this interaction for virus replication or pathogenesis remains unclear [39].

It was shown that deletion of CD2v from the virulent strain BA71 (genotype I) or the strain ASFV-Kenya-IX-1033 (genotype IX) led to the virus attenuation, and these mutants induce protective immunity against infection with a parental or heterologous virus [8,21]. In addition, it has been shown that several natural ASFV isolates with reduced virulence, are non-hemadsorbing, which indicates a link between the function of CD2v and the attenuation of the virus [40,41,42,43]. Thus, two non-pathogenic isolates from Portugal, OURT88/3 and NH/P68, contain frame shift mutations in the EP402R gene, which as a result leads to the absence of a full-sized CD2v protein [40,44,45]. Moreover, recently, an attenuated Latvian field isolate Lv17/WB/Rie1 was found, which did not show hemadsorption. Analysis of the EP402R gene of this isolate showed the deletion of a single nucleotide that led to the formation of a truncated protein [43,45]. On the contrary, CD2v deletion in Malawian strain Lil-20/1 (genotype VIII) [16] or Georgia 2010 isolate (genotype II) [22] did not change the virulence of the ASF virus. Thus, CD2 deletion can have different effects on the virus depending on the ASFV strain used.

To assess the function of CD2v on the replication of virulent ASFV isolates in cell cultures and in domestic pigs, a recombinant virus ΔCongo-v_CD2v containing the deletion of the EP402R gene was obtained. As we have previously shown, a pair of ASFV Congo strains (virulent K49 and attenuated KK262) is a promising model for studying functional genomics and protective immunity against ASF [20,24]. Analysis of the growth kinetics of ΔCongo-v_CD2v in vitro in swine macrophage cell cultures showed no significant differences in virus titers compared to the parental Congo-v virus. Moreover, the analysis of the virulence of the recombinant virus in vivo did not reveal significant differences in the course of the disease, clinical signs or mortality for groups of animals infected with the parental or recombinant virus. However, in animals infected with ΔCongo-v_CD2v, there was a delay in the onset of the disease and viremia. Previously, we also showed that replication of homologous attenuated ASF Congo-a virus with CD2v gene deletion in vivo was significantly reduced compared to the parental Congo-a [20]. In the case of a virulent homologue of the Congo virus with CD2v deletion, the delay in the onset of viremia may also be associated with a lower level of replication in vivo.

Interestingly, our data are consistent with the data obtained in the experiment involving the Malawi Lil-20/1 isolate (genotype VIII) [16]. CD2v deletion in this isolate did not cause changes in growth characteristics in swine primary macrophage cultures; however, the onset of viremia in animals infected with ΔCD2v mutant was significantly delayed (by 2–5 days), and mean viremia titers were reduced by about 10,000 times 5 days after infection [16]. The authors suggested that the observed delay in the generalization of ΔCD2v mutant infection is most likely a direct consequence of a defect in early replication. It is possible that the viremia of many pathogenic ASFV isolates is largely associated with the erythrocytes. The association of ASF-infected cells with erythrocytes may be a mechanism that highly virulent ASF strains use to spread the virus to other organs and also, possibly, to maintain high-virus titers in the blood for further infection of other animals.

However, there are examples of pathogenic non-hemadsorbing ASFV isolates [46]. In addition, it was shown that the deletion of CD2v in the ASFV Georgia 2010 isolate did not significantly change the virulence of the virus [22]. At the same time, the viremia values did not differ from those detected in animals infected with the parental virus.

An attempt was made to improve the experimental vaccine strain ASFV-G-Δ9GL by additional deletion of the EP402R gene [23]. It is important to note that ASFV-G-Δ9GL/ΔCD2v induced almost undetectable levels of viremia during the inoculation of pigs, but could not protect them from infection with the parental virulent ASFV-Georgia, while ASFV-G-Δ9GL provided reliable protection during challenge [23]. Therefore, deletion of CD2v is not associated with decreased virulence in the ASFV Georgia isolate.

Contradictory data on the effect of CD2 protein on the virulence of various strains may be due to the fact that other ASFV genes can functionally compensate for the loss of function of the EP402R gene. However, it is currently not known which ASFV genes may be involved in this mechanism. One of the candidates for this role may be the genes of multigene families (MGF). The essential roles of MGF proteins in in vivo infections, virulence and immune escape have been demonstrated repeatedly [47,48,49,50]. It should be noted that strains BA71V and ASFV-Kenya-IX-1033 contain deletions of some genes of MGF110, MGF360 and MGF505, in contrast to ASFV strains K49, Georgia 2010 and Malawi Lil-20/1 [29,37,51,52,53,54]. This issue requires a more detailed study in the future.

The results presented here confirm that direct extrapolation of the results obtained as a result of deletions of individual genes in a particular strain of the virus to other viral isolates should be avoided. Moreover, the result of genetic modification of the ASF virus can be unpredictable, and in each case, it requires careful experimental verification.

## 5. Conclusions

The EP402R gene (encoding the CD-like protein) is one of the genes whose deletion can lead to a reduction in the virulence of the ASF virus. The CD2v protein is responsible for mediating hemadsorption and participates in the formation of a protective immune response. Deletion of the EP402R gene led to the attenuation of various strains of the ASFV, although the degree of attenuation varies among different isolates. In this paper, we have shown that the deletion of the EP402R gene from the genome of a high-virulent Congo isolate did not change either the virulence of the virus or its ability to replicate in the swine macrophage cell cultures in vitro. However, the onset and the mean value of viremia in animals infected with ΔCongo-v_CD2v were significantly delayed. Thus, deletion of the CD2 gene in different isolates of the ASFV has a different effect on the virulence of the virus, depending on its genetic background.

## Figures and Tables

**Table 1:** Fever, pig survival and viral load in pigs infected with 10^3^ TCID50 ASFV Congo-v or ΔCongo-v_CD2v.

Group	No of Animals	Mortality		Fever		Viral Load in Blood at 3 dpi (log 10 TCID50/mL)	Viral Load in Blood at 7 dpi (log 10 TCID50/mL)	Viral Load in Organs (log 10 TCID50/mL)
		%	TTD (SE)	%	TTF (SE)			
ΔCongo-v_CD2v	8	100	8.5 (0.46)	100	5.5 (0.32)	2.5–4	8.25–8.75	6–8
Congo-v	5	100	7.8 (0.37)	100	4 (0)	7.5–8.5	8.5–8.75	6–7.75
Control	5	0	0	0	0	Neg	Neg	Nt

TTD, Mean time-to-death in days post-challenge, with SE in parentheses. TTF, Mean time-to-fever in days post-challenge, with SE in parentheses. Neg—Negative. Nt—Not tested.
